# Supplementary material for: A web-based atlas for exploring post-transcriptional regulation in the archaeon Sulfolobus acidocaldarius
Source: mSystems. 2025 Dec 11;11(1):e01485-25. doi: 10.1128/msystems.01485-25 (PMC12817955; doi:10.1128/msystems.01485-25)
Supplement: Supplemental material — Figures S1-S5 and legends to the supplemental tables and files. [file msystems.01485-25-s0004.docx]

**Supplementary Figures and Legends**

**A Web-Based Atlas for Exploring Post-Transcriptional Regulation in the archaeon *Sulfolobus acidocaldarius***

Michel Brück^1^, Michael Daume^1,2^, Lennart Randau^1,3*^, José Vicente Gomes-Filho^1*#^

**Affiliations:**

^1^ Faculty of Biology, Philipps-Universität Marburg, Marburg, Hessen, 35039, Germany

^2^ WACKER, Munich, Bavaria, Germany

^3^ Center for Synthetic Microbiology, Philipps-Universität Marburg, Marburg, Hessen, 35039, Germany

**Notes**

José Vicente Gomes-Filho and Lennart Randau are joint senior authors.

*The authors declare no conflict of interest.*

#Address correspondence to José Vicente Gomes-Filho

gomesfil@staff.uni-marburg.de

**
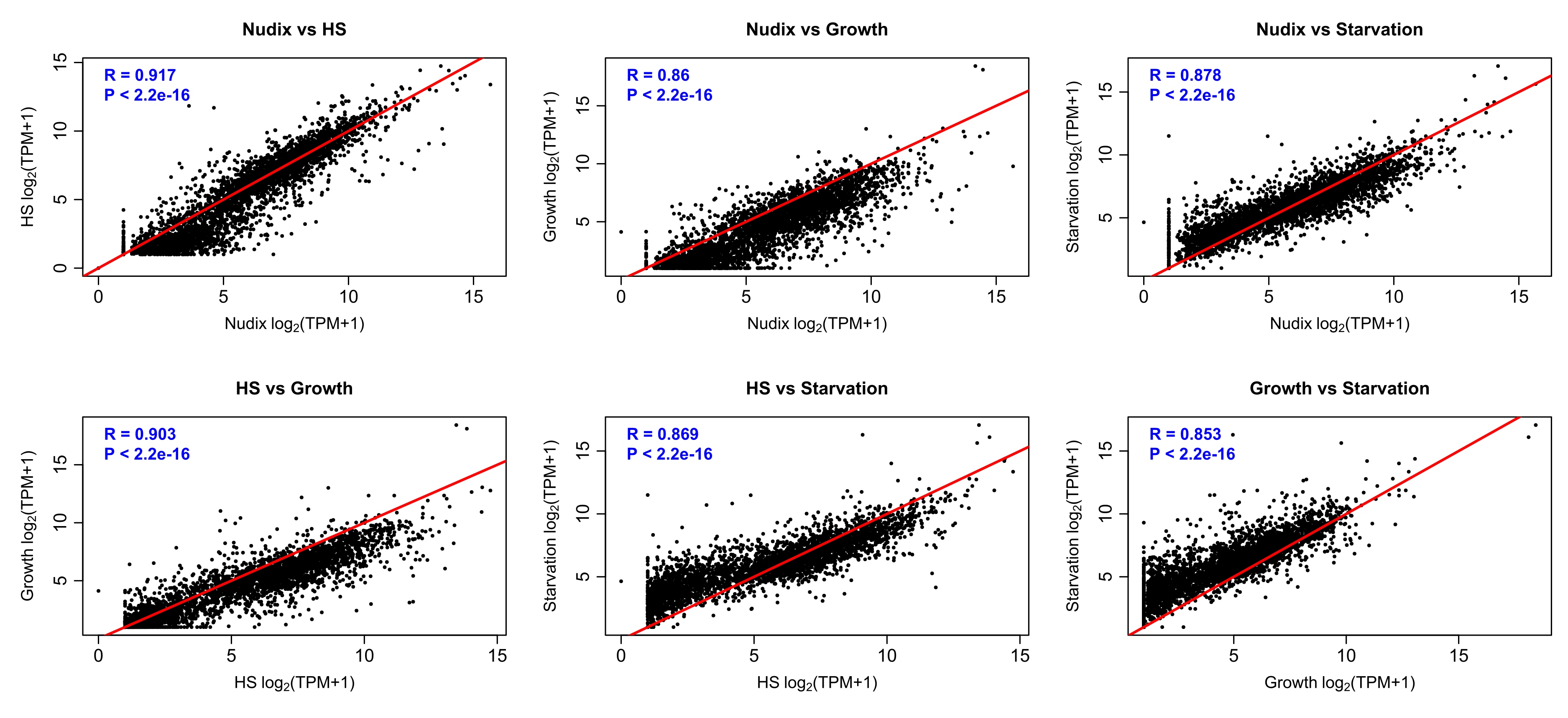
**

**Figure S1:** **Pairwise scatter plots showing log_2_-transformed TPM values of all transcripts between reference datasets from multiple experimental setups.** Red diagonal lines indicate equivalent expression levels. Pearson correlations were consistently high across comparisons (R = 0.853~0.917, p < 2.2e^−16^). These results confirm that global expression profiles are highly reproducible across datasets prepared from standard growth conditions but using different library preparation and sequencing strategies (Table S1). The identifiers “Growth”, “Nudix”, “HS”, and “Starvation” refer to the control datasets from the following experiments: (i) temperature, pH, and growth phase variation; (ii) NUDIX proteins knockout (34); (iii) post-heat stress (86 °C); (iv) post-nutrient limitation, respectively.

**
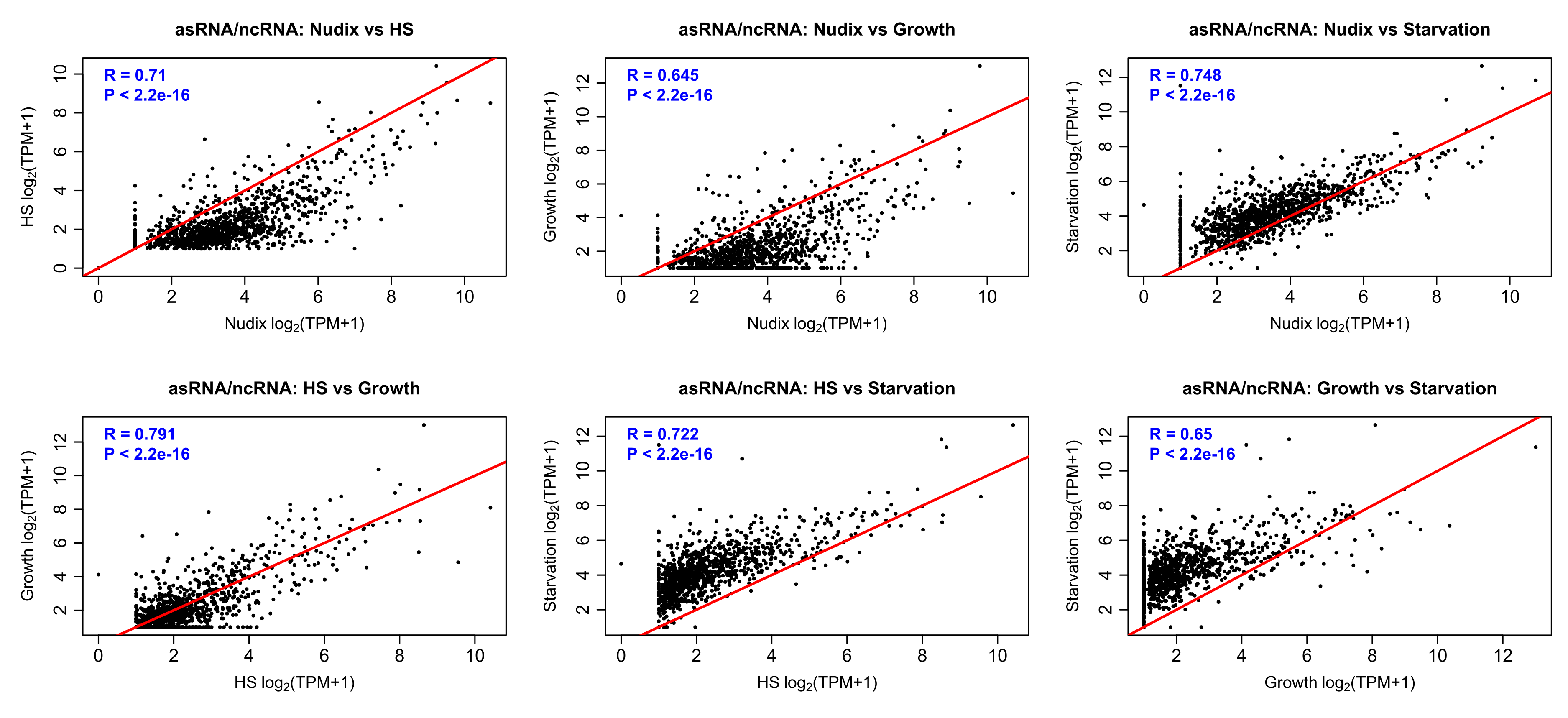
**

**Figure S2: Pairwise scatter plots showing log_2_-transformed TPM values of detected asRNAs/ncRNAs between reference datasets from multiple experimental setups.** Red diagonal lines indicate equivalent expression levels. Limiting the analysis to only asRNAs and ncRNAs reduced the correlations (R = 0.65–0.79 | p-value < 2.26e^-16^), reflecting an increased variability in the detection of these typically shorter and lower-abundance transcripts. Nevertheless, the moderate-to-strong correlations observed for ncRNAs/asRNAs across experiments demonstrate that their detection is reproducible and not simply an artifact of a particular sequencing method (Table S1). The identifiers “Growth”, “Nudix”, “HS”, and “Starvation” refer to the control datasets from the following experiments: (i) temperature, pH, and growth phase variation; (ii) NUDIX proteins knockout; (iii) post-heat stress (86 °C); (iv) post-nutrient limitation, respectively.


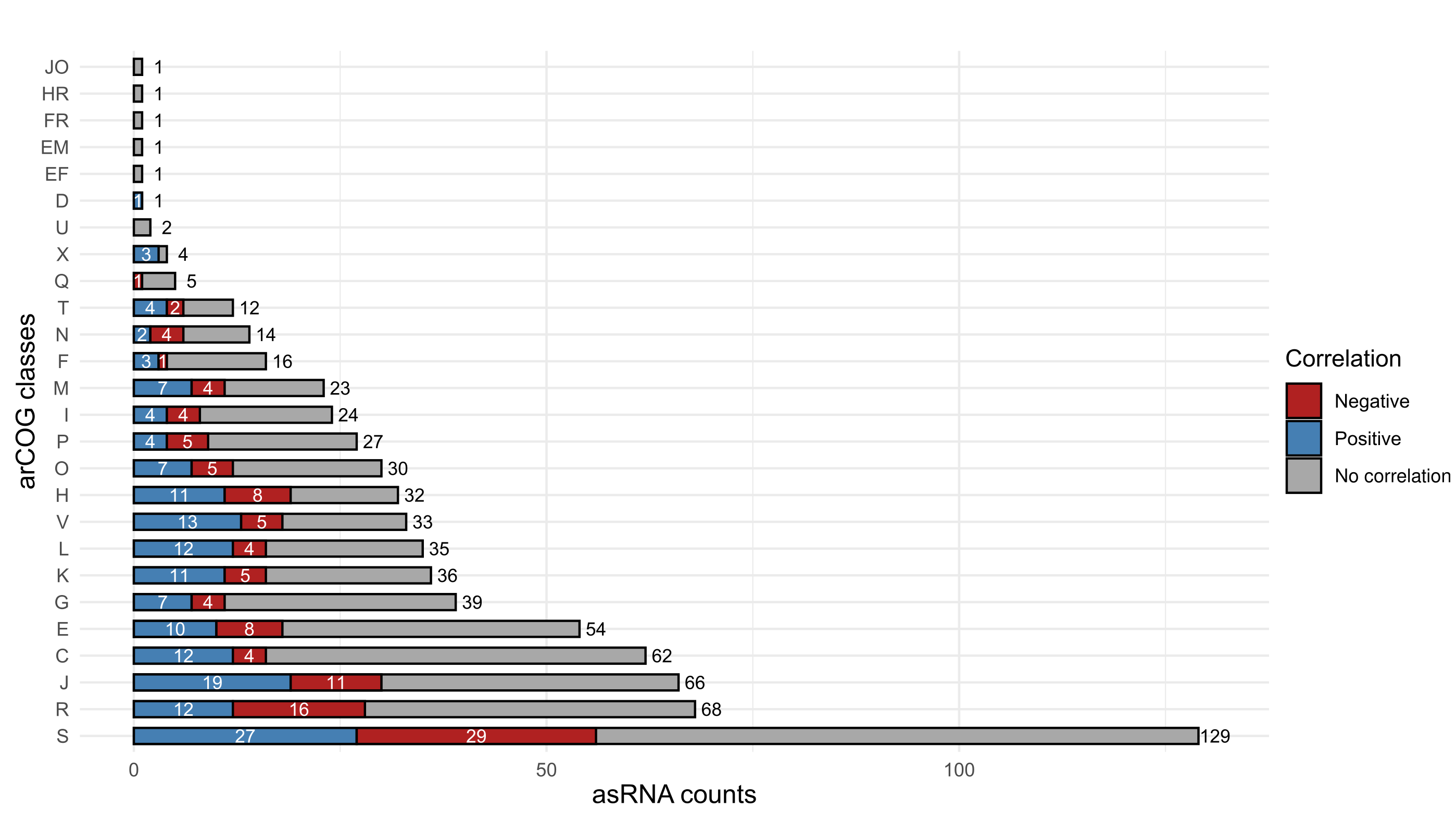


**Figure S3: Distribution of arCOG functional categories among asRNA/mRNA pairs.** Each horizontal bar represents the total number of asRNA/mRNA pairs for the respective category. Colored segments represent pairs with significant correlation. Red indicates negative correlations (PCC ≤ -0.5), suggesting opposing expression trends between the antisense RNA and its cognate mRNA, while blue indicates positive correlations (PCC ≥ 0.5), reflecting coordinated expression. Grey segments represent pairs that are not correlated. The presence of potential antisense regulation is broadly distributed across arCOG categories. The category with the highest counts is (S) unknown function, while increased representation is found in classes such as translation (J) and energy production (C). Remaining categories: General function prediction only (R), Amino acid transport and metabolism (E), Carbohydrate transport and metabolism (G), Transcription (K), Replication, recombination and repair (L), Defense mechanisms (V), Coenzyme transport and metabolism (H), Posttranslational modification, protein turnover, chaperones (O), Inorganic ion transport and metabolism (P), Lipid transport and metabolism (I), Cell wall/membrane/envelope biogenesis (M), Nucleotide transport and metabolism (F), Cell motility (N), Signal transduction mechanisms (T), Secondary metabolites biosynthesis, transport and catabolism (Q), Mobilome: prophages, transposons (X), Intracellular trafficking, secretion, and vesicular transport (U), Cell cycle control, cell division, chromosome partitioning (D), and combined functional categories (JO, HR, FR, EM, EF).


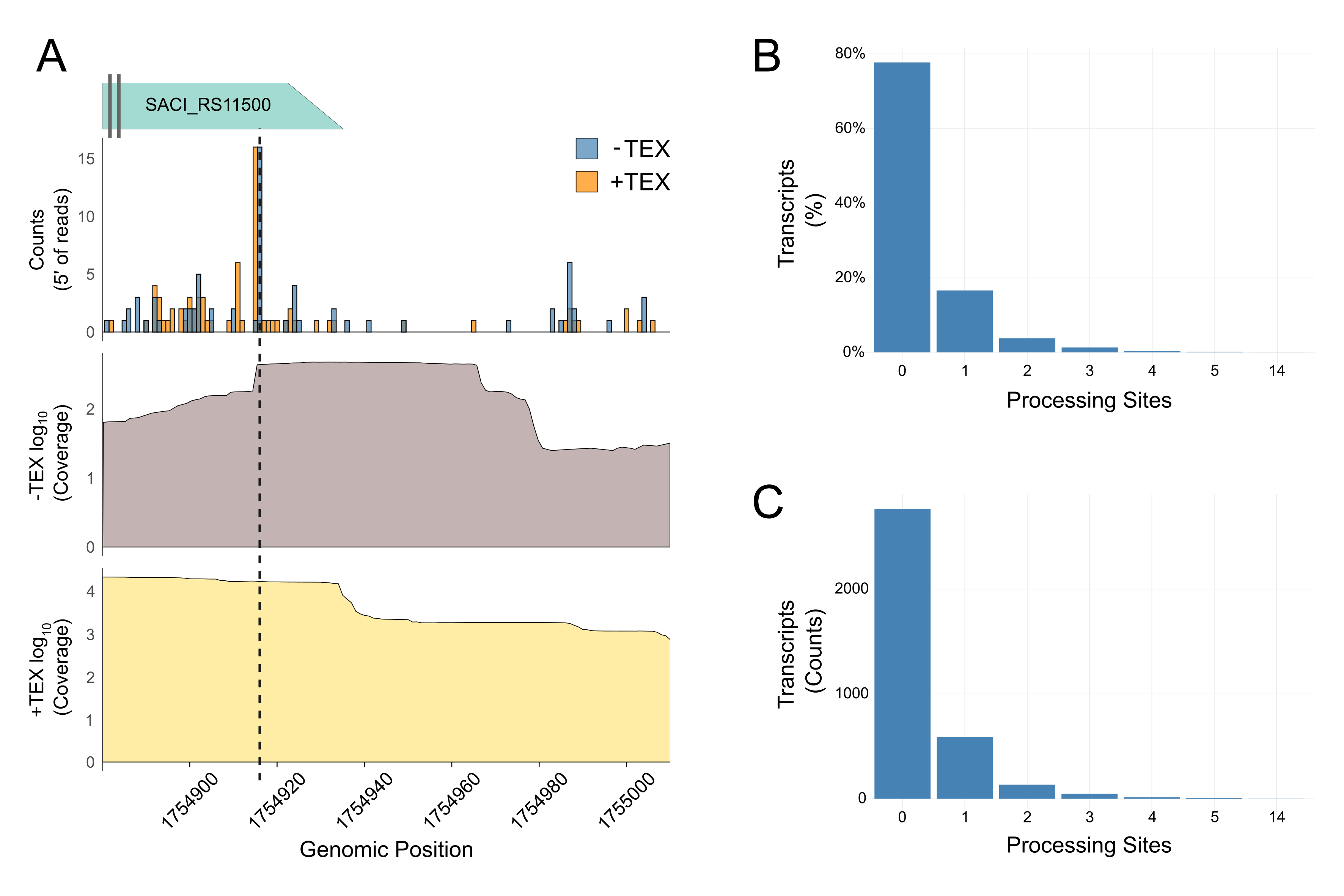


**Figure S4:** **Analysis of the transcript processing site motifs identified with dRNA-seq.** (A) Mapping of 5′ ends at the 3′ end of the gene SACI_RS11500 reveals one TPS possibly associated with the production of 3′-UTR derived ncRNAs. Bar plots show normalized 5′ end read counts from +TEX/–TEX libraries. Additionally, coverage plots of log_10_(Normalized Reads) from -TEX (dark gray) and +TEX (light yellow) show peak depletion at the positions where TPSs are identified. (B) Distribution and accumulation of TPSs per transcript, represented as %. (C) Distribution and accumulation of TPSs per transcript, represented as raw counts.


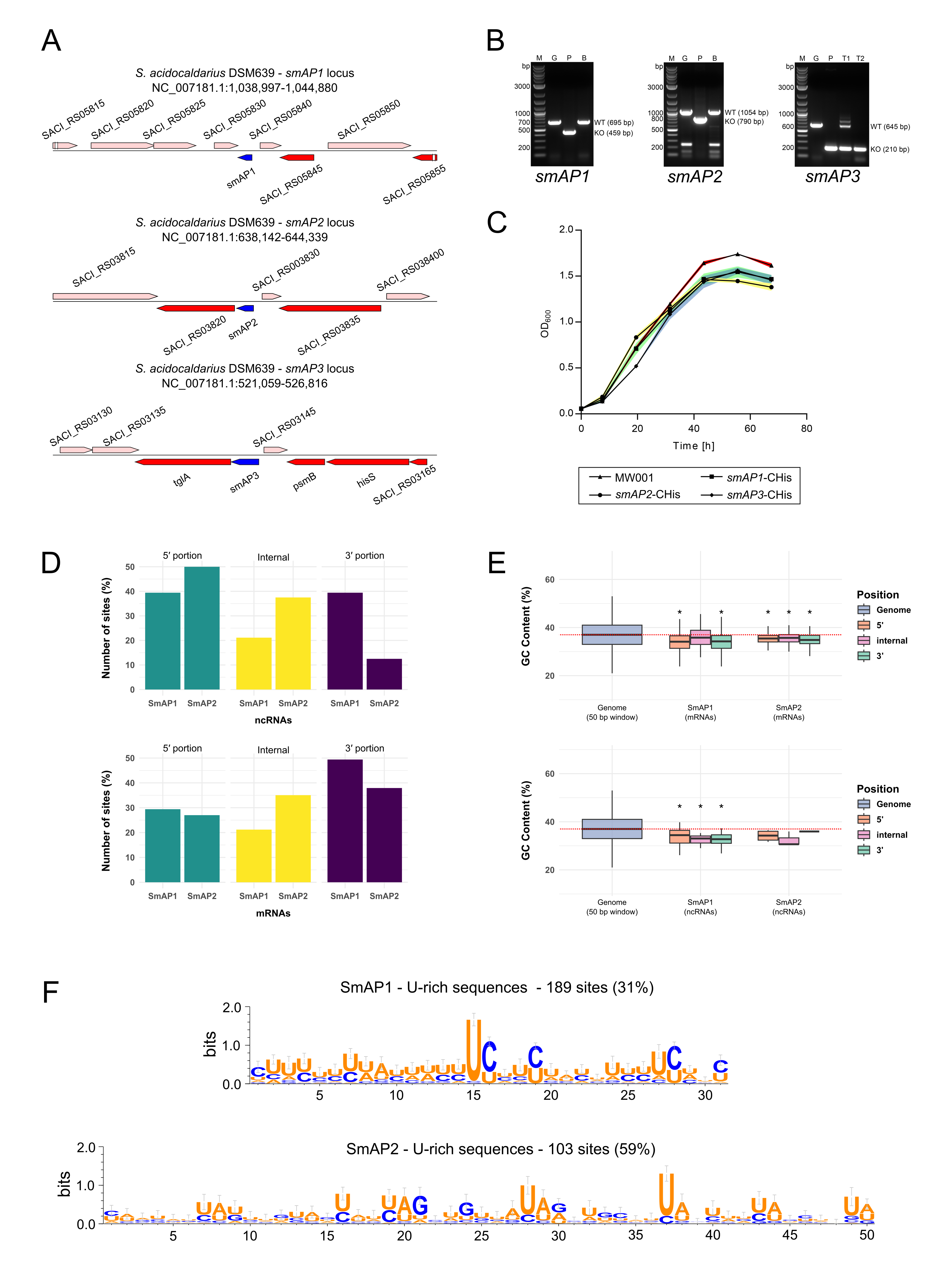


**Figure S5: Loci organization, knockout attempts, and binding properties of SmAPs.** (A) The *smAP1-3* loci. Blue colored arrows: the *smAP* genes. Salmon colored arrows: forward-strand genes. Red colored arrows: reverse-strand genes. (B) Colony PCRs of the *smAP* loci show bands representing the wild-type locus (WT) or the *smAP* deleted locus (KO). Genomic DNA (marked G) of *S. acidocaldarius* and the pCRISPR plasmids (marked P) were used as negative and positive controls, respectively. Representative background colonies (marked B) of the *smAP1* and *smAP2* deletion attempts display WT bands. Two types of transformants (T1/T2) were obtained for the *smAP3* deletion attempt: T1 shows the WT and KO bands as well as additional upper bands, whereas T2 displays a single band for the *smAP3* deleted locus. (C) The growth behavior of genomically His-tagged SmAP strains was compared to the *S. acidocaldarius* DSM 639 MW001 reference strain. Error bars (color-filled area) demonstrate the standard deviation of three technical replicates. (D) positional biases of SmAP1-2 binding. (E) GC content and localization on the binding sites of SmAP1-2 on mRNAs and ncRNAs were evaluated using the Mann-Whitney U test. Significant differences (p < 0.05) between the genomic GC content average and specific classes are shown as * above each boxplot. The horizontal dashed red line indicates the average genomic GC content (37%) of *S. acidocaldarius.* (F) Distinct U-rich sequences for the bound RNAs of SmAP1 and SmAP2 were detected using the MEME suite (51).

**Supplementary Files – Legends:**

File S1 – *Sulfolobus acidocaldarius* DSM639 curated annotation. GFF file containing the most up-to-date RefSeq annotation and the transcripts identified in this study.

File S2 – Differential expression analysis using DESeq2 of all analyzed conditions

File S3 – Transcript processing sites positions and sequences in their vicinity (+10 and -10 nucleotides from the TPS position)

**Supplementary Tables – Legends**

Table S1 – All sources of publicly available RNA-seq data, associated publication (if available), and conditions.

Table S2 – Table of all mRNAs that have at least one associated asRNA

Table S3 – List and specification of each plasmid, strain, and oligos used in this work.
